# Exploring the Complexity of Cortical Development Using Single-Cell Transcriptomics

**DOI:** 10.3389/fnins.2018.00031

**Published:** 2018-02-02

**Authors:** Hyobin Jeong, Vijay K. Tiwari

**Affiliations:** Institute of Molecular Biology, Mainz, Germany

**Keywords:** epigenetics, neurogenesis, development, neocortex, stem cells

## Abstract

The developing neocortex in the mammalian brain is composed of multiple cell types including apical progenitors (AP), basal progenitors (BP), and neurons that populate three different layers, the ventricular zone (VZ), the subventricular zone (SVZ), and the cortical plate (CP). Despite recent advances, the diversity of the existing cell populations including those which are differentiating and mature, their biogenesis and the underlying gene regulatory mechanisms remain poorly known. Recent studies have taken advantage of the rapidly emerging single-cell technologies to decode the heterogeneity of cell populations at the transcriptome level during cortical development and their molecular details. Here we review these studies and provide an overview of the steps in single-cell transcriptomics including both experimental and computational analysis. We also discuss how single-cell genomics holds a big potential in future for brain research and discuss its possible applications and biological insights that can be achieved from these approaches. We conclude this review by discussing the current challenges in the implementation of single-cell techniques toward a comprehensive understanding of the genetic and epigenetic mechanisms underlying neocortex development.

## Deciphering the gene regulatory network underlying development of neocortex using single-cell genomics

The mammalian brain is one of the most complex organs in the body and plays a fundamental role in higher cognitive function (Striedter, 2005). During brain development, the transition of proliferative and multipotent neuroepithelial cells to fully differentiated neurons is called neurogenesis (Urban and Guillemot, 2014). The neurogenesis mainly occurs between embryonic day (E) 11–17 in mouse and gestational week (GW) 8–28 in human (Malik et al., 2013; Taverna et al., 2014; van den Ameele et al., 2014). During this period, neuroepithelium transforms into three different layers including the ventricular zone (VZ), the subventricular zone (SVZ), and the cortical plate (CP) by the sequential events of differentiation (Gotz and Huttner, 2005). Each of the germinal zones is known to be composed of distinct cell types such as apical progenitor cells (AP), basal progenitor cells (BP), and neurons, whose location of mitosis, polarity, and proliferative potential are different (Taverna et al., 2014). Especially, APs include three subtypes such as neuroepithelial cells, derivative apical radial glia (aRG) which express astroglial markers, and apical intermediate progenitors (aIPs) which undergo one round of symmetric neurogenic division. BPs can be further categorized into proliferative basal radial glia (bRG) and neurogenic basal intermediate progenitors (bIPs) whose diversity and composition determine the rate of neuron production and cortical expansion across the species (Florio and Huttner, 2014; Taverna et al., 2014; Dehay et al., 2015). Those progenitor cells differentiate into neurons and constitute the diverse laminar (L1–L6) and areal identities in the cortical plate as a spatiotemporal manner to establish specialized function and neuronal circuit formation (Franco and Muller, 2013; Jabaudon, 2017). During this process, some of the neural progenitors in the germinal zones are differentiating and migrating into the CP in the early stage of neurogenesis, while some of them are still dividing and proliferating until the later time point of neurogenesis. In addition, while they are neurogenic at the early stages of cortical development, they gradually switch to astrogliogenesis in the later stages. This shows that the cell fate commitment of the progenitor cells is highly dynamic and tightly regulated. How many cell fates exist during neurogenesis and how such dynamic cell fate changes are programmed in the gene regulatory network within individual cells is not well-understood.

Current technological advances in single-cell genomics enabled us to isolate individual cells from complex tissues and explore their molecular profiles at the single cell level, which offers the possibility to characterize the cellular heterogeneity and subpopulations (Yuan et al., 2017). Recently, these technologies were implemented to investigate multiple cell types of neural progenitors and mature neurons generated during neurogenesis (Poulin et al., 2016; Telley et al., 2016). In this mini-review, we introduce current workflow in single-cell genomics, biological insights obtained by single-cell neurogenesis studies, and future challenges in the application of single-cell technologies toward a comprehensive understanding of the genetic and epigenetic mechanisms at the single-cell resolution.

## Current workflow of single-cell technologies implemented in the neurogenesis research

**Figure 1:**
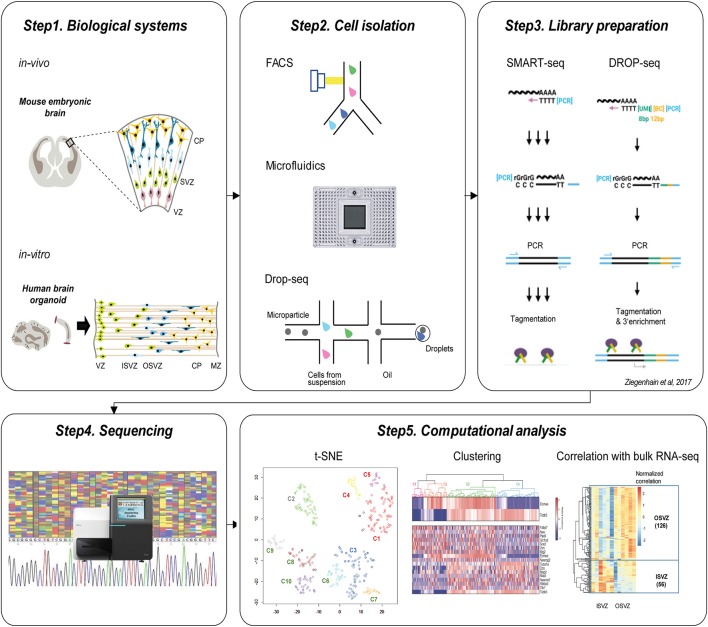
Current workflow of single-cell technologies to study cortical development. Step1. Biological systems to study brain development. Upper panel shows *in vivo* mouse embryonic brain and below panel indicates *in vitro* human brain organoid which is commonly used for the single-cell neurogenesis studies. Step2. Cell isolation methods. Individual cells can be isolated using FACS, Microfluidic ChIP, or Drop-seq approaches. Step3. Library preparation. The common protocols include polyA+ mRNA capture, reverse transcription, cDNA amplification using PCR, and tagmentation. Step4. Sequencing of the library. Step5. Computational analysis. After the preprocessing of sequencing reads, visualization using t-SNE, unsupervised clustering, and correlation analysis with bulk RNA-seq is followed to identify subtypes of cells and characterize their identities.

Current workflow of single-cell genomics is organized in the set of steps: defining the biological system, appropriate isolation of relevant single cells, sequencing library preparation, high-throughput sequencing, and computational analysis (Figure 1).

The two very popular biological systems to investigate cortical development using single-cell genomics have been embryonic cortical tissues and brain organoids. (Figure 1, Step1) For example, single-cell studies have been performed in E13.5 and E14.5 cortex from mouse brain (Fan et al., 2016; Telley et al., 2016) and micro-dissected cortex from 14 to 16 GW and 16 to 18 GW from human fetal brain (Camp et al., 2015; Pollen et al., 2015; Table 1). As an alternative method to overcome the limited accessibility to the fetal human tissues, researchers have developed 3D *in vitro* culture “brain organoid” using human pluripotent stem cells, in which cells self-organize into complex structures. In this technology, inductive signaling molecules mimic endogenous patterning drive dorsal and ventral forebrain differentiation which generate proliferative ventricular-like zones containing neural stem cells that produce a multilayered cortical-like structure expressing markers of deep- and superficial-layer neurons (Di Lullo and Kriegstein, 2017). The brain organoid imitates the features of the developing human brain *in vivo* (Kelava and Lancaster, 2016), and it has been successfully used for single-cell transcriptome studies. For example, Camp et al. profiled single-cell transcriptome from 333 cells of human brain organoid and found that human cerebral organoids recapitulate gene expression programs of fetal neocortex development (Camp et al., 2015). Quadrato et al. profiled transcriptome from 80,000 single cells from 31 human brain organoids and showed that organoids could generate a broad diversity of cell types that reflect endogenous classes (Quadrato et al., 2017). Given their ability to recapitulate the cell diversity of the cortical development, the brain organoids in combination with single-cell techniques will continue to provide useful information on human neurogenesis and neurodevelopmental disorders (Bershteyn et al., 2017; Table 1).

**Table 1:** Application of single-cell technology to neurogenesis research.

**System**	**Species**	**Study**	**Age/Stage**	**Target layer**	**Cell isolation**	**Library generation**	**Number of cell**	**Reads per cells**	**Genes per cells**	**Computational analysis**
*In vivo* neurogenesis	Mouse	Telley et al., 2016	E14.5	Isochronic cohorts of newborn VZ cells	FACS	SMARTer ultra low RNA kit for the C1 system (Takara Clontech, #634833)	272 cells	0.6 million	4,726	t-SNE, SCDE
	Mouse	Fan et al., 2016	E13.5	NPCs	FACS	SMARTer ultra low RNA kit for illumina sequencing (catalog no. 634936)	65 cells	20 million	5,909	PAGODA
	Human	Pollen et al., 2015	GW 16–18(14–16 wpc)	VZ, SVZ	Microfluidic; Fluidigm C1	SMARTer ultra low RNA kit (catalog no. 63495, PT5163-1)	393 cells	2.5 million	3,100	t-SNE, ConsensusClusterPlus, EMCluster, DESeq2
	Human	Camp et al., 2015	12–3 wpc	neocortex	Microfluidic; Fluidigm C1	SMARTer ultra low RNA kit for Illumina (Clontech)	226 cells	2–5 million reads	2,744	Correlate with bulk RNA-seq, Monocle TF correlation network analysis
*In vitro* neurogenesis	Human	Camp et al., 2015	Days 33, 35, 37, 41, and 65, iPSC-derived	Cerebral organoid	Microfluidic; Fluidigm C1	SMARTer ultra low RNA kit for illumina sequencing (Clontech)	333 cells	2–5 million reads	4,218	t-SNE, correlate with bulk RNA-seq
	Chimpenzee	Mora-Bermudez et al., 2016	7 organoids (45–80 days)	Cerebral organoid	Microfluidic; Fluidigm C1	SMARTer ultra low RNA kit for the Fluidigm C1 system	344 cells	1 million	2,730	t-SNE, SCDE, correlate with bulk RNA-seq
	Human	Bershteyn et al., 2017	WT (2 individuals), MDS (3 individual), 5, 10, 15 weeks of differentiation	Cerebral organoid	C1 single-cell auto prep integrated fluidic circuit (IFC, Fluidigm)	SMARTer ultra low RNA kit	469 cells	–	–	PCA, ConsensusClusterPlus R
	Human	Quadrato et al., 2017	3–6 month	Cerebral organoid	Drop-seq	Drop-seq	82,291 cells	0.1 million	~1,300	Seurat

To isolate individual cells (Figure 1, Step2), Fluorescence-activated cell sorting (FACS) (Fan et al., 2016; Telley et al., 2016) and microfluidic systems (Fluidigm C1) (Camp et al., 2015; Pollen et al., 2015; Mora-Bermudez et al., 2016; Bershteyn et al., 2017) have been most widely applied. FACS isolate cells of interest using the targeted cell-surface markers so that it provides the possibility to enrich for fluorescently-labeled cells of interest as described before (Telley et al., 2016). The microfluidic system such as Fluidigm C1 uses the microfabrication techniques and microfluidic chambers to isolate single-cells (Saliba et al., 2014). On the other hand, Drop-seq was currently developed as microdroplet system using microfluidic technologies to isolate single cells in aqueous droplets in a non-aqueous suspension which serve as individual nanoliter-scale aqueous reaction chambers for reverse transcription of PCR (Macosko et al., 2015; Poulin et al., 2016). Drop-seq was recently implemented for the study of 80,000 cells from human brain organoid (Quadrato et al., 2017). It seems that for hundreds to thousands of cells, FACS or microfluidic system (Fluidigm C1) is recommended for cell isolation, while to scale-up to thousands to tens of thousands of cells, Drop-seq technique is suitable (Poulin et al., 2016) though it has limitation of low gene-per-cell sensitivity compared to other scRNA-seq methods (Ziegenhain et al., 2017).

Following single cell isolation, cells are lysed and the RNA is captured for reverse transcription into cDNA to construct sequencing library. Previous single-cell genomics applied in the neurogenesis research mostly implemented template-switch-based protocols including Smart-seq and DROP-seq (Figure 1, Step3) (Camp et al., 2015; Pollen et al., 2015; Fan et al., 2016; Mora-Bermudez et al., 2016; Telley et al., 2016; Bershteyn et al., 2017; Quadrato et al., 2017). In case of Smart-seq, commercially available Smart-seq kit (Clontech) is used to generate full-length double-stranded cDNA which is converted into sequencing libraries by tagmentation (Nextera, Illumina) (Ziegenhain et al., 2017). Smart-seq2 protocol is similar to Smart-seq which generates full-length libraries, but it had improved reverse transcription, template switching, and pre-amplification to increase yield and length of cDNA libraries from single cells (Picelli et al., 2013; Ziegenhain et al., 2017). In Drop-seq, a flow of beads are suspended in lysis buffer and a flow of a single-cell suspension is brought together in a microfluidic chip, which generates nanoliter-sized emulsion droplets. Here each bead contains covalently bound oligo-dT primers carrying a unique molecular identifier (UMI) and a unique, bead-specific barcodes. UMI is a barcode of the individual molecule to estimate the number of transcribed molecules that is independent of amplification biases (Stegle et al., 2015), while bead-specific barcode provides the information of cell-of-origin (Macosko et al., 2015). Following cell lysis, their mRNA gets attached to the oligo-dT-carrying beads, and then as droplets are broken, cDNA and library are generated for all cells in parallel.

Prepared libraries undergo sequencing using next-generation sequencing platforms such as Illumina Hi-Seq and Nextseq (Figure 1, Step4). Both single-end (Chu et al., 2016; Xu et al., 2016) and paired-end (Telley et al., 2016) library preparation are used for the single-cell transcriptomic analysis. For the special purpose of investigation of transcript isoforms, paired-end sequencing is suitable to quantify multiple isoforms with high confidence. In terms of sequencing depth, the recent single-cell transcriptomics from the neurogenesis research sequenced 0.1–5 million reads per cell (Table 1). To get a saturated gene detection, 1 million reads per cell is generally recommended (Svensson et al., 2017). However, the sequencing depth has to be decided based on the purpose, as not all studies need to saturate detection but some of them more focus on the finding of the new cluster of cells which requires a large number of cells rather than high sequencing depth. For example, Pollen et al. performed down-sampling analysis from the 301 single-cells of developing cerebral cortex and found that 0.05 million reads per cell is sufficient for unbiased cell-type classification and biomarker identification (Pollen et al., 2014).

Following sequencing, an extensive computational analysis is performed including read alignment, quantification, visualization of data, unsupervised clustering, and differential expression analysis to interpret these large-scale data sets (Figure 1, Step5). After the read alignment and quantification using Tophat (Kim et al., 2013), STAR (Dobin et al., 2013), Cufflinks (Trapnell et al., 2012), or Kallisto (Ntranos et al., 2016), the low-quality cells with small library size or high portion of mitochondrial reads need to be excluded from downstream analysis. Toward the visualization of single-cell transcriptomes at the collective level, most studies in past implemented Principal component analysis (PCA) and t-SNE to obtain the overview and structure of subpopulations (Poirion et al., 2016). For the unsupervised clustering, ConsensusClusterPlus R (Wilkerson and Hayes, 2010), EMCluster (Jung et al., 2014), SC3 (Kiselev et al., 2017), SNN-Cliq (Xu and Su, 2015), SCUBA (Marco et al., 2014), BackSPIN (Zeisel et al., 2015), and PAGODA (Fan et al., 2016) provide methods to identify the subpopulation from the single-cell transcriptome profiles. Following clustering, DESeq2 (Love et al., 2014), SCDE (Kharchenko et al., 2014), and MAST (Finak et al., 2015) are used to identify differentially expressed genes between clusters. Pseudotime is another important concept in the computational analysis of single-cell transcriptome, which estimates the cells' progress through the transition. The computational tools like TSCAN (Ji and Ji, 2016), Monocle (Trapnell et al., 2014), Waterfall (Shin et al., 2015), Sincell (Julia et al., 2015), Oscope (Leng et al., 2015), and Wanderlust (Bendall et al., 2014) provide *in silico* defined pseudotime for each single-cell during the cell fate transition.

To gain the first glimpse into the characteristic of single-cell clusters, typically the expression of marker genes such as proliferation, neuronal onset, and neuronal differentiation/maturation genes (Telley et al., 2016) and/or correlation with bulk-cell transcriptome profiles is integrated. For example, Camp et al. performed unsupervised clustering of 226 single-cells from human embryonic neocortex, and examined the characteristics of each clusters (Camp et al., 2015) using the correlation with existing bulk-cell RNA-seq profiled from cortical layers (VZ, ISVZ, OSVZ, and CP; Fietz et al., 2012) and FAC-sorted subpopulations (aRG, bRG, and N; Florio et al., 2015). Furthermore, Mora-Bermudez et al. performed single-cell RNA-seq from 344 cells of Chimpanzee cerebral organoids and compared each cell cluster with bulk-RNA-seq from germinal layers of the human embryonic brain (Fietz et al., 2012; Mora-Bermudez et al., 2016). These abovementioned steps are the most widely followed in the current single-cell studies to decode heterogeneity in cell populations during cortical development.

## Novel biological insights into cortical development using single-cell technologies

Current single-cell genomics studies (Table 1) have provided unprecedented biological insights into the cellular diversity and its molecular code which was difficult to obtain using previous approaches. For example, a recent study performed single-cell RNA-seq of isochronic VZ cells after 6, 12, 24, and 48 h of birth (Telley et al., 2016). Following this, computational pseudotime modeling which projects each cell into the differentiation trajectory identified sequential waves of gene expression patterns, perturbation of which restricted formation of proper neuronal layers. Furthermore, epigenetic factors such as Kdm3a (lysine demethylase 3A) and MeCP2 (Methyl CpG binding protein-2) belonged to different sequential waves, suggesting that distinct epigenetic players contribute at defined steps of neurogenesis.

Interestingly further, single-cell transcriptome analysis in combination with an unsupervised clustering has not only been able to dissect cellular heterogeneity but also characterize molecular details of the identified subpopulations of cells. For example, a previous study revealed that the most significant aspect of heterogeneity was originating from genes associated with neuronal maturation and growth, which is closely tied to the spatial organization of their expression patterns across three layers (VZ, SVZ, and CP) of the developing cortex (Fan et al., 2016). In another study, two different radial glial cell populations oRG and vRG were separated based on the single-cell transcriptome profiles and it further allowed a thorough investigation of differences in the gene expression profiles between these two cell populations (Pollen et al., 2015). For example, the key regulators such as HOPX and PTPRZ1 were found to be differentially expressed between oRG and vRG and may guide future studies aimed to decipher the differential transcriptome underlying identity of oRG and vRG cells.

Another considerable point of single-cell RNA-seq analysis is that the identification of similarities and differences of cell populations between *in vivo* and *in vitro* neurogenesis, or between species. For instance, single-cell transcriptomes from *in vitro* human brain organoids could faithfully reconstruct genetic and cellular networks involved in germinal zone organization, neural progenitor cell (NPC) proliferation, and NPC-to-neuron differentiation *in vivo* (Camp et al., 2015). In this study, over 80% of genes that were differentially expressed across the fetal cortex lineages have similar expression profiles in organoid and fetal cerebral cortex (Camp et al., 2015). Furthermore, in a study comparing AP populations between species, about 12% of the genes specific to AP or neurons in both human and chimpanzee were found not specific to these cell types in the mouse, implying an involvement of certain specific developmental mechanisms during the development of the primate cerebral cortex (Mora-Bermudez et al., 2016). Altogether, these examples vouch for the strong and unprecedented discovery power that single-cell transcriptomics has conferred researchers in the field of cortical development.

## Challenges in single-cell technologies for cortical development research

Despite exciting advances in single-cell genomics, there are several challenges toward deciphering the gene regulatory network and epigenetic mechanisms of cell fate specification during neurogenesis at the single cell level (Poulin et al., 2016). Current single-cell transcriptome studies in neurogenesis research implemented dissociation of cells from the tissue which involves external physical stress (Liu and Trapnell, 2016). In addition, this procedure requires the removal of cell-cell contacts. Since niche microenvironment and cell-cell adhesion are also means of signal transduction, it is not clear how much the loss of these properties influences the transcriptome at the single cell level. To reduce these issues, alternative single-cell transcriptome techniques such as *in situ* sequencing (Ke et al., 2013) and Fluorescent *in situ* sequencing (FISSEQ) (Lee et al., 2015) could be considered for future neurogenesis studies.

Furthermore, current single-cell transcriptome only gives a snap-shot of the analyzed cell at the time of capture. These transcriptome data also have a large sparsity with a very high proportion of genes that show zero read counts (Vallejos et al., 2017). This zero count can come from biological reasons (a transient state where a gene is not expressed) as well as technical reasons such as dropout events and read depth of sequencing. Therefore, it is not fully clear how much of the single-cell transcriptome data and resulting clusters are influenced by any of these variables. To reduce the bias from the technical issue, more effort to increase capture efficiency is needed for library preparation (Liu and Trapnell, 2016). In parallel, thorough normalization of data and quality control processes are needed to address the technical issues come from sparsity of the data or cell cycle phase transition (Vallejos et al., 2017). Also, it is essential to combine dual measurements from the same cell that allows transcriptome analysis simultaneous to another readout of the cellular state. Along these lines, new techniques combining live-cell imaging and single-cell sequencing (Lane et al., 2017), or electrophysiology and single-cell sequencing (Cadwell et al., 2016), which can track cellular state in parallel with genome-wide gene expression profiles are increasingly getting popular and should be applied to the studies of cortical development.

The recent decade has shown that epigenetic mechanisms are critical for gene regulatory programs underlying cell-fate changes during development. Recently, single-cell ATAC-seq (Buenrostro et al., 2015) was applied to neurogenesis study (Preissl et al., in review) to measure chromatin accessibility at the single cell level. However, many other single-cell epigenomics methods including DROP-ChIP (Rotem et al., 2015), scRRBS (Guo et al., 2013), and scHi-C (Ramani et al., 2017) to measure chromatin landscape, DNA methylatome and higher-order chromatin structures at the single cell level remained to be applied to study brain development. Furthermore, those protocol can be combined into single-cell multi-omics technique such as scMT-seq (Hu et al., 2016), scTrio-seq (Hou et al., 2016), and scNMT-seq (Clark et al., in review). Current single-cell epigenome technology has the limitation of low coverage of genome so that the clustering of cells are biased by easily profiled genomic regions. If this limitation is improved, these single-cell epigenomic technologies will enable us to decipher epigenetic control of cortical development and its contribution to the sequential waves of transcriptional changes that underlie neurogenesis. In addition, single-cell epigenomics also holds potential to identify new cell subpopulations during cortical development that were not detected by single-cell transcriptome analysis.

Given that the field of single-cell genomics is relatively new, the researchers also encountered challenges in having universally accepted and robust pipelines for the computational analysis of single-cell datasets. Compared to conventional bulk RNA-seq analysis, single-cell RNA-seq analysis requires more rigorous quality control and normalization to minimize the bias arising from low capture efficiencies and confounding factors like cell cycle state changes. Although individual tools specialized for the analysis of single-cell data are increasingly available (Poirion et al., 2016), a standard pipeline that includes quality controls, normalization, clustering, finding the identity of clusters and differential expression analysis should be established to provide robust and comparable results between different laboratories. Also, novel analysis ideas which can find new insight from the data, or improve the quality of unsupervised clustering need to be developed continuously.

Importantly further, it is also possible to use the existing single-cell transcriptome profiles from neurogenesis *in vivo* and *in vitro* to analyze splicing, non-coding RNA species, and intronic transcripts. While most of the single-cell transcriptome profiling protocols so far employed poly-A selection, a subset of the non-coding RNAs which contain poly-A tail can be assessed. The intronic reads from nascent RNAs can be measured from the single-cell transcriptome to study splicing and actual rates of transcription (Gaidatzis et al., 2015). Recently developed approaches including BRIE (Huang and Sanguinetti, 2017), WemIQ (Zhang et al., 2015), and SingleSplice (Welch et al., 2016) will help analyzing alternative splicing from the existing single-cell transcriptomes of neurogenesis. Given that alternative splicing (Vuong et al., 2016) and non-coding RNA-mediated gene regulation (Yao et al., 2016) are known to be important for neurogenesis, investigation of splicing regulation, non-coding RNA, and nascent RNA expression from the existing single-cell transcriptomes will provide novel insights into the heterogeneity of cell populations and molecular programs underlying cortical development.

## Conclusions and perspectives

Recent single-cell transcriptome studies allowed novel discoveries on various aspects of cortical development including sequential waves of gene expression, cellular heterogeneity, and comparative analysis of cell populations across embryonic stages, species, and origins (*in vitro*/*in vivo*). Future studies should invest more effort to improve library preparation protocols to increase the molecular capture efficiency to reduce the bias from the technical issue. Also, simultaneous assessment of cellular state such as live cell imaging and electrophysiology in addition to gene expression profiling at the single-cell level need to be considered. Moreover, efforts should be made to measure single-cell transcriptome without detachment of cells from cortex and organoids to allow proper assessment of cellular states and transcriptional programs underlying neurogenesis. These assessments will also remain incomplete unless complemented by a systematic investigation into the epigenetic landscape of single-cells using technologies such as DROP-ChIP, scMT-seq, and scTrio-seq. Those multi-omics approaches will enable the generation of mechanistic models relating genetic/epigenetic variation and transcript expression dynamics in neurogenesis (Macaulay et al., 2017). Additionally, development of robust and universally accepted computational pipelines is required to obtain more conclusive biological findings and their comparability across different laboratories. At the same time, existing single-cell genomics data can be further analyzed by novel computational methodologies to profile alternative splicing, non-coding transcripts, and nascent RNAs. Importantly, all of these comprehensive single-cell genomics analysis should be performed at various stages of cortical development for the comprehensive understanding of cellular subpopulations. Altogether, with these advances, we will get closer to decoding the complexity of cell types and underlying gene regulatory network during cortical development.

## Author contributions

Both authors listed have made a substantial, direct and intellectual contribution to the work, and approved it for publication.

### Conflict of interest statement

The authors declare that the research was conducted in the absence of any commercial or financial relationships that could be construed as a potential conflict of interest.
